# Sublethal Effects of Polystyrene Nanoplastics on the Embryonic Development of *Artemia salina* (Linnaeus, 1758)

**DOI:** 10.3390/ani13193152

**Published:** 2023-10-09

**Authors:** Martina Contino, Greta Ferruggia, Stefania Indelicato, Roberta Pecoraro, Elena Maria Scalisi, Antonio Salvaggio, Maria Violetta Brundo

**Affiliations:** 1 Department of Biological, Geological and Environmental Sciences, University of Catania, Via Androne 81, 95124 Catania, Italy; greta.ferruggia@phd.unict.it (G.F.); stefania.indelicato@phd.unict.it (S.I.); roberta.pecoraro@unict.it (R.P.); elenamaria.scalisi@unict.it (E.M.S.); mariavioletta.brundo@unict.it (M.V.B.); 2 Zooprophylactic Institute of Sicily “A. Mirri”, Via Gino Marinuzzi, 3, 90129 Palermo, Italy; antonio.salvaggio@izssicilia.it

**Keywords:** nanoplastics, polystyrene, *Artemia salina*, embryotoxicity, biodistribution, oxidative stress, apoptosis

## Abstract

**Simple Summary:**

Due to ubiquitous pollution and the consequent climatic changes facing the scientific community, it is imperative to evaluate the possible consequences related to the exposure of organisms to environmental pollutants, nowadays predominated by micro and nanoplastics. Aquatic environments are, among environmental matrices, those most susceptible to the dispersion of such harmful substances. The purpose of the present study was to assess the exposure to polystyrene nanoplastics of species at the base of the food chain in aquatic ecosystems with a focus on embryonic development. The occurrence of embryo morphological and metabolic changes such as the stimulation of apoptosis emphasizes the potential ecotoxicological effects of nanoplastics.

**Abstract:**

Currents, wave motion, solar radiation, and abrasion are mechanisms responsible for the degradation of large plastic artifacts and contribute to the dispersion of micro and nanoplastics into aquatic ecosystems, which are, currently, the most dangerous threats due to their invisibility and persistence. The present work evaluated the possible lethal and sublethal effects of amino-modified polystyrene nanoplastics (nPS-NH_2_) with diameters of 50 nm and 100 nm on *Artemia salina (A. salina)*, an organism at the base of the trophic chain of the aquatic system, using a widely used model for the analysis of embryotoxicity from environmental pollutants. For this purpose, after evaluating the biodistribution of nanoplastics in the body of the tested animals, several endpoints such as anomalies, apoptosis, and ROS production were assessed. In addition, particular attention was dedicated to evaluating the correlation between toxicity and the particle size tested. The results reported that, despite the absence of a lethal impact, several sublethal effects involving gut and body size malformations, as well as the enhancement of apoptosis and oxidative stress in relation to an increase in tested concentration and a decrease in nanoparticle size.

## 1. Introduction

In both salt and freshwater ecosystems (seas, oceans, lakes, and rivers), the trophic web is finely regulated by interspecific predation relationships that maintain the balance between the communities of producers and primary and secondary consumers [1,2,3]. The base of the aquatic food chain is dominated by plankton (phytoplankton, bacterioplankton, and zooplankton), which include small organisms that float or move in the surface part of water bodies [4]. Zooplankton, in particular, is characterized by different classes of organisms such as microcrustaceans, copepods, and rotifers that feed on other plankton, playing the role of primary consumers [5]. Zooplankton is used for ecotoxicological assays as a biological indicator to provide information on water quality and to examine the effects of pollutants (metals, drugs, engineered nanoparticles, and pesticides) on biological communities and the balance of ecosystems [6,7,8,9]. *Artemia salina*, commonly known as the sea monkey, is a microcrustacean that lives in hypersaline aquatic ecosystems and is one of the most studied organisms of zooplankton, especially in the embryological field, since its very early developmental stages (nauplii) are sensitive to perturbations of the microenvironment caused by the influence of pollutants [10,11]. Recently, *Artemia* spp. has also been a candidate model for the genotoxicity of emerging pollutants [12,13]. Evidence from ecotoxicological studies on its larvae suggests individual and collective predictive value, suggesting not only possible consequences on individual aquatic organisms but also possible alterations in the physiology and biodiversity of an entire biological ecosystem. Indeed, a decrease in the number of species at the base of the food chain inevitably results in negative stresses for populations at the upper levels of the trophic chain. The scarcity of food in both amount and quality is, in fact, directly correlated with increased competition among individuals and the decreased abundance of a population [14].

The current global landscape is dominated by plastic diffusion and pollution, resulting in negative pressures that have been amply demonstrated in all environmental matrices. These impacts are most pronounced in aquatic ecosystems, where, due to biotic (microorganisms) and abiotic (wave motion, currents, winds, abrasion, UV rays) action, large plastic artifacts (macroplastics) release gradually smaller fragments until they reach a size on the microscale (microliter, 0.1–5 mm) and nanoscale (<100 nm) [15,16,17,18]. Nanoplastics are defined as nanoparticles (nanospheres, nanowires/nanotubes, or nanofilms) of plastic, which include any synthetic or semisynthetic organic polymer with at least a size between 1 and 100 nm [19] or less than 1 μm [20]. Their composition can be heterogeneous and include different types of polymers such as polyethylene (PE), polypropylene (PP), polyvinyl chloride (PVC), polyethylene terephthalate (PET), polystyrene (PS), and nylon (PA). Polystyrene (PS), particularly (C_8_H_8_)_n_, is a high-molecular-weight aromatic polymer characterized by styrene monomers that is employed to produce containers, trays, and disposable tableware (cutlery, glasses, plates, and bowls) as well as for the synthesis of creams in the field of cosmetology [21]. From the start of its production, annual global production has increased exponentially, and it is estimated that 21 million tons of PS are produced annually, which corresponds to 7.1% of all plastic produced worldwide [22]. In addition, PS has been found in all major environmental matrices, but its highest concentrations were observed in waters near the most industrialized and populated areas of the planet [23].

Because their density is lower than that of water, nanoplastic fragments tend to float or remain in the upper part of the water column (the optimal habitat for zooplankton), indicating that the first biological system has been exposed to plastic pollution. Moreover, the specific gravity of small plastics is similar to that of algae, belonging to phytoplankton and constituting the main diet for zooplankton [24]. Indeed, it has been shown that zooplankton, during its grazing activity, cannot distinguish micro and nanoplastics from food, reducing the consumption of organic particles derived from primary producers [25,26]. The entry of plastic nanoparticles into zooplankton organisms is reflected in the impact on large aquatic organisms that ingest zooplankton and indirectly absorb microplastics, causing biomagnification and bioaccumulation [27,28,29]. Indeed, the negative effects of the accumulation of plastics as well as other emerging contaminants such as heavy metals (arsenic, cadmium, and lead) in sensitive target organs such as the gut have been recorded [30]. In addition, microplastics alone or in combination with metal contaminants are known to induce oxidative stress and apoptotic phenomena in aquatic species such as goldfish, zebrafish, and *Cyprinus carpio*, as well as in terrestrial species such as mice or chicken [31,32,33,34,35,36,37,38,39].

*A. salina* plays a crucial role in the contamination of species of interest for human consumption such as fish and crustacean larvae due to its employment as a feeder in the aquaculture industry [40,41,42,43]. Aquaculture waters are also contaminated with a plethora of xenobiotics such as resilient plastics. Microplastics have been found in both mariculture and freshwater areas around the world [44]. The predominant plastic types are fibers, followed by microspheres, fragments, films, and granules [45,46,47]. It has also been shown that areas designated for farming are more contaminated due to their location, usually near industrialized areas, and the intensive use of drugs and chemicals can transport plastic fragments [48,49]. It seems clear, then, how the pollution of aquaculture products is a danger to humans and that monitoring the dynamics of the biomagnification of plastics is imperative to adopt interventions to prevent and protect humans and environmental health.

Given the crucial role of *A. salina* as an animal model, whose data can be translocated to other species, this in vivo study sought to elucidate the possible lethal and sublethal effects of 50 nm and 100 nm polystyrene nanoplastics at increasing concentrations following acute (48 h) exposure, focusing on their biodistribution and the possible correlation between toxicological profile and size. An independent study was conducted to investigate the possible influence of the tested nanoplastics on the hatching rate, while a second experiment focused on nauplii to assess vitality rate, possible malformations, apoptosis, and ROS overexpression.

## 2. Materials and Methods

### 2.1. Preparation of Solution

Most studies in the literature have focused on the toxicity of micrometer-sized plastic fragments, but biotic and abiotic degradative processes originate nanofragments and nanospheres that can more easily encounter even the smallest organisms in aquatic ecosystems [50,51]. For this reason, polystyrene nanospheres (nPS-NH_2_) with diameters of 100 nm and 50 nm, purchased from Sigma-Aldrich (St. Louis, MO, USA), were selected. The former was fluorescent in green/orange (481/644 nm excitation/emission) while the latter was fluorescent in blue (358/410 nm excitation/emission). Nanospheres are chemically modified with an amine functional group on the surface; therefore, they are positively charged. They are widely distributed in nature compared to virgin plastics because of chemical modifications made during the manufacturing process of the products to facilitate bonding with other polymers or with additive molecules, making the polymers more stable and durable [52]. These nPS-NH_2_ exhibit a density of 1.03–1.07 g/mL. The work concentrations were selected based on previous studies conducted on other animal species belonging to zooplankton and used as models for ecotoxicological assays, including different species of *Artemia* [53,54]. Working solutions with concentrations of 10 mg/L and 20 mg/L were prepared from the stock solution. According to APAT and IRSA-CNR (2003) [55], the nPS-NH_2_ were dissolved in previously prepared artificial salt water (ASPM) with a solution containing (per liter) NaCl = 26.4 g; KCl = 0.84 g; CaCl_2_•H_2_O = 1.67 g; MgCl•6H_2_O = 4.6 g; MgSO_4_•7H_2_O = 5.58 g; NaHCO_3_ = 0.17 g; H_3_BO_3_ = 0.03 g. The salts were dissolved in distilled water by stirring. The resulting solution was used following the verification of the pH (7.4). All working solutions were sonicated for 3 min to avoid the aggregations of particles and maintain the monodispersion and stability of nanoplastics [56].

### 2.2. Hatching Assay

The hatching assay was performed by placing 50 mg of cysts into several beakers containing 250 mL of solutions with increasing concentrations of nPS-NH_2_ (50 and 100 nm) diluted in ASPM. The cysts were soaked at a temperature of 25 ± 1 °C for 48 h, with continuous aeration and illumination (3000–4000 lx). At the end of the time, the samples were observed under the stereomicroscope to calculate the hatching rate.

### 2.3. Embryo Exposure

An acute toxicity test was performed according to standardized protocols with some modifications [55]. Next, 100 mg of *A. salina* cysts were placed in a beaker containing 500 mL of ASPM for 48 h at a temperature of 25 ± 1 °C with continuous aeration and illumination (3000–4000 lx). The exposure was performed for 48 h on nauplii at stages II–III, and the phases of embryonic development were considered crucial for the formation of healthy juvenile organisms; in addition, these stages are more sensitive to the presence of environmental pollutants. Once the cysts had hatched, nauplii at the second/third stage of development were selected using a stereomicroscope (Leica EZ4, Leica Mycrosystem, Buccinasco, MI, Italy). A nauplium was placed in each well of a 96-well plate into which 200 µL of the test solution was subsequently dispensed. The nauplii were left on display for 48 h without food. In each plate, the last column was used to create an internal control to highlight any possible contamination that could invalidate the results. A plate was considered valid if the viability of the larvae in the internal control was greater than 90%. The negative control was made by exposing nauplii from one plate only to ASPM, while the positive control was made by exposing nauplii to potassium dichromate. At 24 h intervals, different parameters were observed to assess the possible toxic action of the nPS-NH_2_.

### 2.4. Localization of Nanoplastics

At 24 h intervals, nauplii were observed under a light microscope (Nikon eclipse Ci, Nikon Europe B.V., Amstelveen, The Netherlands) and fluorescence microscope (Leica DMLB, Leica Mycrosystem, Buccinasco, MI, Italy) to determine the localization of nPS-NH_2_ during the early stages of embryonic development. Fluorescence images were subjected to analysis using Nis Element 5.20, which provided the fluorescence intensity emitted by the larvae.

### 2.5. Assessment of Viability

Embryo viability was assessed using a stereomicroscope (Leica EZ4). Nauplii were considered dead when, following a 10 s observation, no movement (change of barycenter position and appendage) occurred [57]. The test was considered valid if the mortality of the control group was less than 10% after 48 h.

### 2.6. Malformation Analysis

The morphology of the larvae was evaluated in detail using both light microscopy (using fresh larvae) and scanning electron microscopy (SEM, Coxem EM-30 plus, Coxem, Daejeon, Korea). For SEM investigations, the larvae were fixed in 2.5% glutaraldehyde in PBS for 24 h at 4 °C. Subsequently, larvae were washed in PBS for 10 min and dehydrated with a series of alcohols of increasing concentration (35°, 50°, 70°, 95°, and 100°). The obtained samples were incubated with hexamethyldisyllazine (HDMS, Merk, Dramstadt, Germany) and 100° alcohol (1:1 ratio, *v*/*v*) for 5 min and in pure HDMS for 5 min and left to air dry overnight. Samples were mounted on specific carbon fiber-covered stubs and observed using SEM (Coxem EM-30 plus).

Total body length was assessed through image (acquired by an optic microscope) analysis performed using ImageJ 1.44, calculating the distance between the eye and the tail end.

### 2.7. Analysis of Apoptosis

Cell apoptosis was analyzed using Acridine Orange dye (AO, Invitrogen, Waltham, MA, USA) [58]. Thirty nauplii (per replica) were washed in PBS and incubated with 20 µL of 5 µg/mL AO for 20 min at room temperature. After washing in PBS, the nauplii were observed under a fluorescence microscope (Nikon eclipse Ci). The captured images were analyzed using Nis Element 5.20 software through which regions of interest (ROIs) were created to evaluate the fluorescence intensity of equal areas in different larvae. Mean intensity values less than 15 Arbitrary Units (A.U.) were considered negative (mean intensity of control) and higher values were considered positive [59].

### 2.8. In Vivo Evaluation of ROS

An oxidative stress assessment was performed using the DCFH_2_-DA probe (Sigma-Aldrich), which emits green fluorescence when oxidized [60]. Thirty embryos (per replica) were washed in PBS and exposed to a 10 µM probe for 15 min in the dark at room temperature. Subsequently, the larvae were washed in PBS and observed under a fluorescence microscope (Nikon eclipse Ci). The captured images were analyzed with Nis Element 5.20 software through which ROIs (regions of interest) were created to evaluate the fluorescence intensity of equal areas in different larvae. Mean intensity values less than 15 A.U. (Arbitrary Units) were considered negative (mean intensity of control) and higher values were considered positive.

### 2.9. Statistical Analysis

The data obtained were analyzed using Past 4.0 software after verifying the data distribution (Shapiro–Wilk) and homogeneity of variances (Bartlett) through an ANOVA test, followed by Tukey’s test to assess statistically significant differences between the exposed groups and the control. All data were reported as mean ± standard deviation. The symbol * indicates significant data (*p* < 0.05), while the symbol ** indicates highly significant data (*p* < 0.01). For this experiment, 3 replicates were conducted.

## 3. Results

### 3.1. Hatching Assay

**Figure 1:**
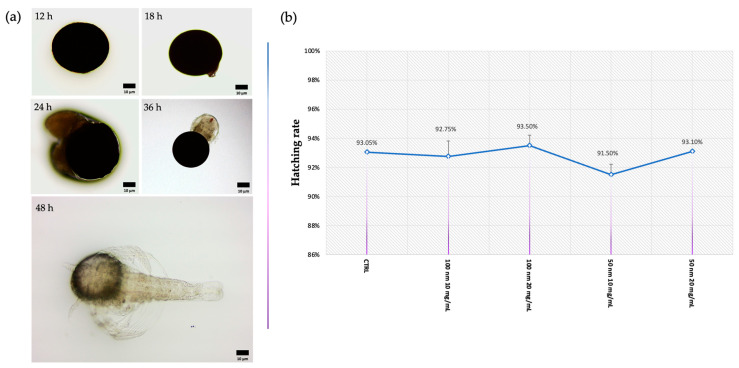
Hatching assay: (**a**) observation of *A. salina* cyst under an optic microscope at various time intervals; (**b**) mean percentage of hatching after exposure to 50 nm and 100 m nPS-NH_2_ at increasing concentrations. No differences were observed between groups.

The normal course of hatching *A. salina* cysts is shown in Figure 1a. Once in contact with water, the cysts begin to rehydrate and resume their metabolism; meanwhile, the embryo inside continues its development (12 h) until the latter begins to rupture the cyst from within (18 h). After 24 h, the embryo begins to emerge, extruding itself from the envelope at 48 h. Statistical analysis showed no difference between the hatching rate of the samples exposed to both types of nPS-NH_2_ and the control group. In fact, the hatching rate in all exposed groups amounted to around 92%, as shown in Figure 1b.

### 3.2. Localization of Nanoplastics

**Figure 2:**
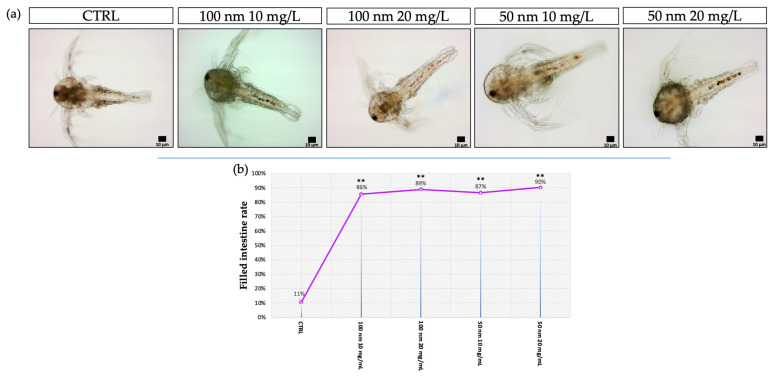
(**a**) Observation of nPS-NH_2_ localization using light microscopy after 48 h of exposure; (**b**) mean percentage of larvae with filled intestine after 48 h of exposure to 50 nm and 100 m nPS-NH_2_ at increasing concentrations. Strong statistical data (** *p* < 0.01) were reported for all exposed groups compared to the control.

**Figure 3:**
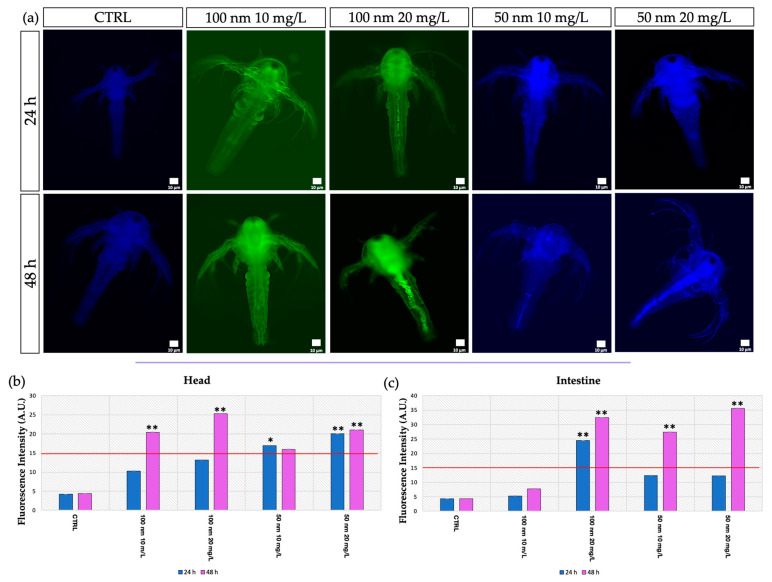
Localization of polystyrene nanoplastics: (**a**) Observation of nPS-NH_2_ localization using fluorescence microscopy at 24 h and 48 h in *A. salina* larvae exposed to 50 nm and 100 m nPS-NH_2_ at increasing concentrations; (**b**) mean of fluorescence intensity of the head, acquired using image analysis with Nis Element. The threshold of the test (15 A.U.) is represented with a red line. Statistical differences are indicated with * (*p* < 0.05) and strong significance of data is indicated with ** (*p* < 0.01); (**c**) mean of the fluorescence intensity of the intestine acquired using image analysis with Nis Element. The threshold of the test (15 A.U.) is represented with a red line. Strong statistical differences are indicated with ** (*p* < 0.01).

At 24 h intervals, the localization of the nPS-NH_2_ was visualized using light and fluorescence microscopy. From an initial observation via light microscopy (24 h), no particles were observed. An accumulation of nanoplastics is evident in the intestine at 48 h, as shown in Figure 2a. A high percentage of larvae that were positive for intestinal aggregations was reported for all exposed groups (86% ± 0.007 for 10 mg/L of 100 nm; 89% ± 0.002 for 20 mg/L of 100 nm; 87% ± 0.007 for 10 mg/L of 50 nm; 90% ± 0.003 for 20 mg/L of 50 nm) compared with the control (11% ± 0.007) (Figure 2b). This result was also evidenced following fluorescence observation and the calculation of fluorescence intensity using image analysis with Nis Element, through which the nPS-NH_2_ were also localized at the head level to 48 h (Figure 3a). In addition, the abundance of fluorescence spots increased in relation to the concentration tested. Regarding the anterior part of the body (head), compared with the control, in which negative larvae show a mean fluorescence intensity of 4.2 ± 0.003 at 24 h and 4.4 ± 0.001 at 48 h, the group exposed to 10 mg/L of 100 nm nanoplastics had a fluorescence intensity of 10.3 ± 0.004 at 24 h and 20.4 ± 0.002 (** *p* < 0.01) at 48 h, while the mean fluorescence intensity following exposure to the concentration of 20 mg/L of the same nanoplastics was 13.2 ± 0.013 at 24 h and 25.3 ± 0.003 (** *p* < 0.01) at 48 h. Finally, larvae exposed to 50 nm nanoplastics had a mean fluorescence intensity of 17 ± 0.002 (* *p* < 0.05) at 24 h and 16 ± 0.005 at 48 h for the lower concentration and a mean fluorescence intensity of 20 ± 0.012 (** *p* < 0.01) at 24 h and 21 ± 0.006 (** *p* < 0.01) at 48 h for the higher concentration. Fluorescence positivity was also recorded in the intestine, mainly after 48 h of exposure to nanoplastics. In detail, compared with the control, in which negative larvae showed a mean fluorescence intensity of 4.3 ± 0.008 at 24 h and 4.4 ± 0.005 at 48 h, the group exposed to 10 mg/L of 100 nm nanoplastics had a fluorescence intensity of 5.3 ± 0.005 at 24 h and 7.8 ± 0.012 at 48 h, while the mean fluorescence intensity following exposure to the concentration of 20 mg/L of the same nanoplastics was 24.5 ± 0.013 (** *p* < 0.01) at 24 h and 32.4 ± 0.003 (** *p* < 0.01) at 48 h. Finally, larvae exposed to 50 nm nanoplastics had a mean fluorescence intensity of 12.4 ± 0.003 at 24 h and 27.4 ± 0.002 (** *p* < 0.01) at 48 h for the lower concentration and a mean fluorescence intensity of 12.3 ± 0.002 at 24 h and 35.6 ± 0.003 (** *p* < 0.01) at 48 h for the higher concentration (Figure 3b,c).

### 3.3. Viability

**Figure 4:**
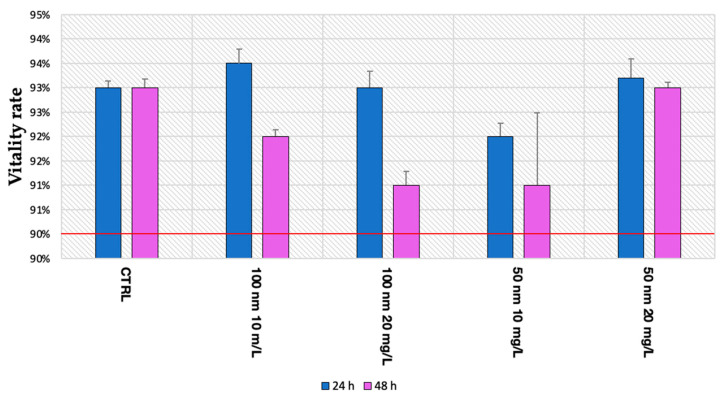
Mean percentage of vitality after exposure to 50 nm and 100 m nPS-NH_2_ at increasing concentrations. No statistical differences were found between groups. The red line indicates the threshold of test validity.

Regarding viability, no statistically significant data were found, as shown in Figure 4. In all samples analyzed, the survival rate was greater than 90% at both 24 h and 48 h. In detail, after 24 h of exposure, the rate of vitality was 93% ± 0.001 in the control group, as well as 93.5% ± 0.002 and 93% ± 0.003 in the larvae exposed to 100 nm nPS-NH_2_ for the concentrations of 10 mg/L and 20 mg/L, respectively, and 92% ± 0.002 and 93.2% ± 0.003 in the groups exposed to 10 mg/L and 20 mg/L of 50 nm nPS-NH_2_, respectively. At 48 h, the vitality rate of the control (93% ± 0.001) did not change; meanwhile, in the other groups, the worsening of the parameter was manifested by a reduction in a few percentage points (92% ± 0.001 for 10 mg/L of 100 nm nPS-NH_2_; 91% ± 0.002 for 20 mg/L of 100 nm nPS-NH_2_; 91% ± 0.014 for 10 mg/L of 50 nm nPS-NH_2_; 93% ± 0.014 for 20 mg/L of 50 nm nPS-NH_2_).

### 3.4. Malformation

**Figure 5:**
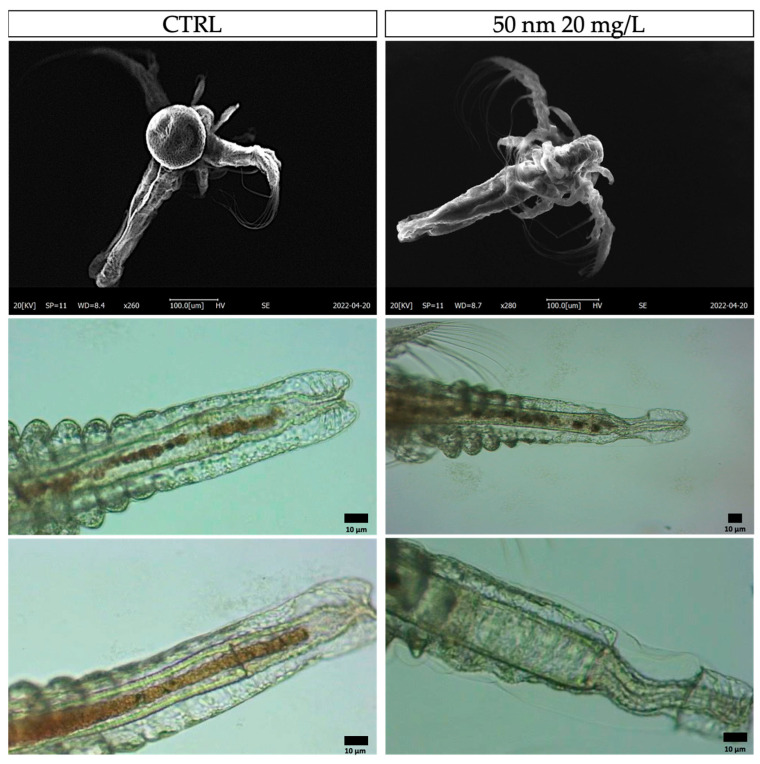
Observation of nauplii intestine constriction under SEM and optic microscope.

**Figure 6:**
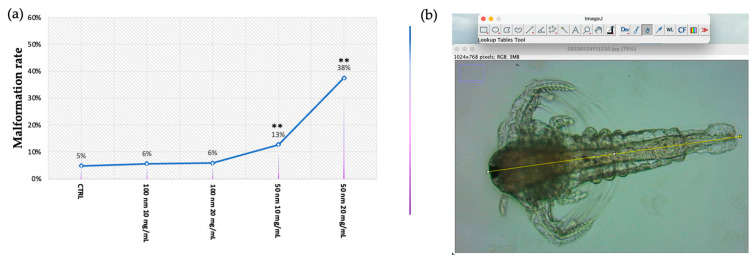
(**a**) Mean percentage of malformed larvae after exposure to 50 nm and 100 m nPS-NH_2_ at increasing concentrations. Strong statistical data (** *p* < 0.01) were reported for larvae exposed to 50 nm nPS-NH_2_ at both concentrations tested; (**b**) analysis of total body length using ImageJ, considering the distance between the eye and final part of the tail.

Despite the absence of lethal effects, in many cases, following exposure to the smaller nPS-NH_2_, the occurrence of some constrictions in the terminal part of the intestine was reported, as shown in Figure 5. Moreover, all samples with this anomaly also had a shorter body than the control. These malformations were found especially in the samples exposed to 50 nm nPS-NH_2_, which were interrelated with an increase in concentration. While the malformation rate in the control group and on larvae exposed to 10 mg/L and 20 mg/L of 100 nm nPS-NH_2_ amounted to 5% ± 0.003, 6% ± 0.001, and 6% ± 0.002, respectively, it corresponds to 13% ± 0.033 and 38% ± 0.035 on larvae exposed to the lower concentration (10 mg/L) and to the higher concentration of 50 nm nPS-NH_2_, respectively (Figure 6a). Through the use of ImageJ, it was possible to calculate the length of the body, which, in the control, was found to be 0.90 ± 0.05 mm, which is similar to the body of samples exposed to 10 mg/L and 20 mg/L of 100 nm nPS-NH_2_ (0.89 ± 0.001 and 0.88 ± 0.02). Meanwhile, in the samples exposed to 10 mg/L and 20 mg/L of 50 nm nPS-NH_2_, it was 0.78 ± 0.012 mm and 0.71 ± 0.015 mm, respectively (Figure 6b).

### 3.5. Apoptosis

**Figure 7:**
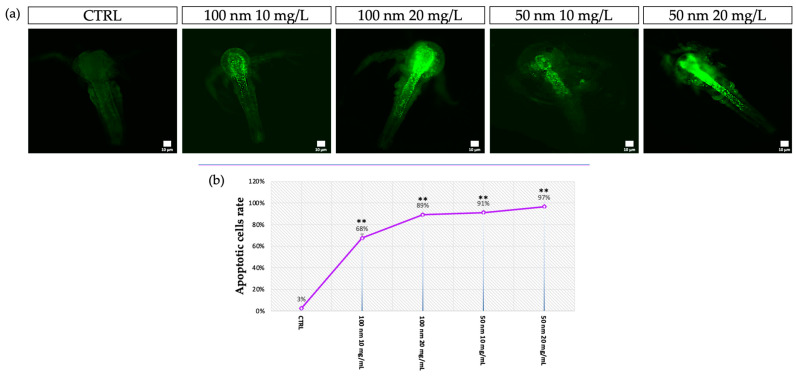
Identification of apoptotic cells using Acridine Orange (AO): (**a**) Observation of larvae under a fluorescence microscope after exposure to 50 nm and 100 m nPS-NH_2_ at increasing concentrations; (**b**) mean percentage of AO-positive larvae after exposure to 50 nm and 100 m nPS-NH_2_ at increasing concentrations. Strong statistical differences (** *p* < 0.01) were reported for all exposed groups compared to the control.

**Figure 8:**
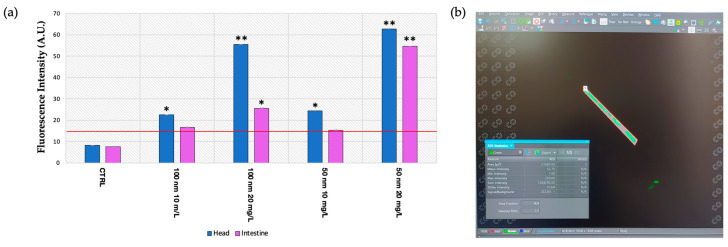
(**a**) Mean of fluorescence intensity acquired using image analysis with Nis Element. The threshold of the test (15 A.U.) is represented with a red line. Statistical differences are indicated with * (*p* < 0.05) and a strong significance of data is indicated with ** (*p* < 0.01); (**b**) examples of image analysis using Nis Element software with the identification of ROIs.

The analysis of apoptosis by staining with Acridine Orange showed the presence of apoptotic cells in the anterior part of the body, especially at the level of the head, in larvae exposed to the lower concentrations. At higher concentrations, however, positivity was also found at the level of the anterior part of the intestine (Figure 7a). From the analysis of the fluorescence image, strong significant data (** *p* < 0.01) were highlighted in all exposed samples compared to the control. Both 50 nm and 100 nm nPS-NH_2_ caused an increase in the rate of cells in apoptosis in relation to the increase in tested concentration (Figure 7b); however, as confirmed by image analysis at the head and gut level (ROIs), the 50 nm nanoplastics caused a greater increase in fluorescence. Compared with the control, in which only 3% ± 0.007 of the larvae were apoptosis-positive (97% negative larvae with a mean fluorescence intensity of 8.2 ± 0.001 for the head and 7.6 ± 0.002 for the gut), the group exposed to the 100 nm nanoplastics had 68% ± 0.035 (with a mean fluorescence intensity of 22.5 ± 0.045 for the head and 16.7 ± 0.056 for the gut) and 89% ± 0.014 positivity (with a mean fluorescence intensity of 55.4 ± 0.023 for the head and 25.6 ± 0.033 for the gut) following exposure to the concentrations of 10 mg/L and 20 mg/L, respectively. Finally, larvae exposed to 50 nm nanoplastics had 91% ± 0.014 (with a mean fluorescence intensity of 24.4 ± 0.065 for the head and 15.5 ± 0.77 for the gut) and 97% ± 0.007 positivity (with a mean fluorescence intensity of 62.7 ± 0.087 for the head and 54.6 ± 0.023 for the gut) following exposure to concentrations of 10 mg/L and 20 mg/L, respectively (Figure 8a,b).

### 3.6. In Vivo Evaluation of ROS

**Figure 9:**
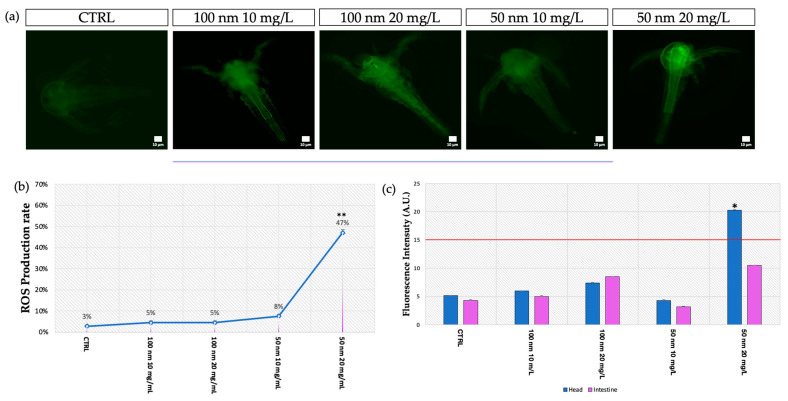
In vivo evaluation of ROS using DCFH_2_-DA: (**a**) Observation of larvae under a fluorescence microscope after 48 h of exposition to 50 nm and 100 m nPS-NH_2_ at increasing concentrations; (**b**) mean percentage of ROS-positive larvae after exposure to 50 nm and 100 m nPS-NH_2_ at increasing concentrations. The symbol ** indicates a strong statistical difference ( *p* < 0.01) between the group exposed to 20 mg/L of 50 nm nPS-NH_2_ and the control; (**c**) mean of fluorescence intensity acquired using image analysis with Nis Element. The threshold of the test (15 A.U.) is represented with a red line. Significant data (* *p* < 0.05) were found for the group exposed to 20 mg/L of 50 nm nPS-NH_2_, in which the fluorescence was localized in the head.

Regarding reactive oxygen species (ROS), the analysis using the DCFH_2_-DA probe (Figure 9a) showed slight positivity (20.3 ± 0.092), for only the heads(47% ± 0.014) of the larvae exposed to the 50 nm nPS-NH_2_ at the highest concentration (20 mg/L) compared to the control group, in which 97% of negative larvae had a mean intensity of 5.2 ± 0.023 on the head (Figure 9b,c).

## 4. Discussion

Plastic pollution has become a major environmental concern due to its ubiquitous presence and degradation into smaller particles, such as nanoplastics, which have attracted increasing attention in recent years because of their potential adverse effects on living organisms. Indeed, research related to the impacts of nanoplastics has evidenced that they can be ingested and accumulated by various organisms, causing toxic effects at the cellular and molecular levels [61]. The purpose of the present work was to evaluate the possible lethal and sublethal effects of nPS-NH_2_ on the embryonic development of *A. salina*, a zooplankton organism that is crucial for the food web in aquatic environments and aquaculture. 

An initial independent experiment was performed on cysts to assess the hatching rate following exposure to the tested nanoplastics. From the data obtained, no delay in hatching was shown between the exposed groups and the control. This could be related to the high resistance of the cysts, which, in the diapause state, exhibit a high tolerance to various external stresses [62]. Indeed, the cysts have the chorion in the outermost layer and the compact, multilamellar embryonic cuticle in the inner layer, isolating the embryo from the outside world and providing only homeostasis and inorganic ion exchange [63].

Given the contact of the nanoplastics with the nauplii at the time of hatching, in the second phase of the experimental design, at 24 h intervals, larvae were monitored to define the location of the nPS-NH_2_ to elucidate their ability to bioaccumulate in this organism. Then, several parameters such as vitality rate, apoptosis, ROS production, and malformations were assessed to analyze possible sub-lethal effects. nPS-NH_2_ were observed mainly in the gut and head of the larvae as demonstrated by fluorescent image analysis. At the head level, while at 24 h only 50 nm nanoplastics were detectable at both concentrations tested, at 48 h, nauplii exposed to all concentrations tested exhibited fluorescence positivity. In contrast, an accumulation of nanoplastics in the intestine was observable at 48 h for both concentrations of the 50 nm nanoplastics and as early as 24 h for the higher concentration of the 100 nm nanoplastics. The fact that the smaller nanoplastics at 48 h were evident at the head and not in the gut could probably indicate the faster absorption and biodistribution of the larger nanoplastics. The possibility of nanoplastics being absorbed and biodistributed in the body has also been proposed by other studies such as the one conducted by Bergami et al. [64], who showed polystyrene uptake as early as 12 h after exposure. Moreover, in this study, the authors noted the release of only part of the aggregates, in the form of fecal pellets, after 48 h. Therefore, it can be assumed that the remaining part was absorbed in the intestine and distributed to various organs. In addition, several zooplankton species (*Daphnia magna* and *Artemia franciscana*) are known to uptake plastic fragments with a size range of up to 30 µm—a food-like diameter [65]. In this context, the kinematics of uptake in relation to time and nanoplastic size needs further investigation.

The present study did not report any lethal effects. Existing literature has generally signaled sublethal effects related to exposure to polystyrene even at different concentrations and sizes (6–10 µm at a concentration of 2 mg/L) [66,67,68]. Lethal effects have been observed with the use of particles (10 µm) of polyethylene (which, to date, seems to be the most toxic type of plastic in the short term [69]) or nanoparticles for chronic exposure (14 d) to a concentration of 20–50 µg/mL [70,71]. Again, further studies will have to be performed in order to assess the actual exposure conditions of zooplankton in nature. Aquatic ecosystems are simultaneously contaminated by different types of micro and nanoplastics that are different in both type and size. Thus, their physical and chemical behavior in relation to the presence of additives and other pollutants remains to be clarified [72]. Co-exposure could result in a synergistic, antagonistic, or no effect on aquatic organisms. For example, association with organic matter (eco-corona), favored by the presentation of positive charges on the surface, worsens the toxicological profile of nanoplastics, which are highly toxic to zooplankton [73].

Nevertheless, in this experiment, some sublethal effects, which could cause long-term toxicity and negative consequences, were described, in particular, an increase in the apoptotic phenomenon mainly at the level of the head and intestine, organs where the nPS-NH_2_ were mainly concentrated. As hypothesized for polystyrene microplastics [54], the induction apoptosis was also correlated in a dose-dependent manner.

Regarding oxidative stress, the results of the present study showed fluorescence spots obtained using probe oxidation only at the head level of organisms exposed to the 50 nm nPS-NH_2_ and at the higher concentration. This is probably related to the size of the nPS-NH_2_. In fact, it is known that a smaller sphere diameter results in a greater chance of reaching the cytoplasm of cells and causes imbalances in metabolism with altered enzymes and transcripts [53,66]. These data suggested the probable existence of a size and concentration range that, within which, antioxidant systems with which microcrustaceans are equipped, including catalase, superoxide dismutase, and glutathione peroxidase, can eliminate radical molecules [74,75].

Finally, growth inhibition with a reduction in total body length has been highlighted, especially in larvae exposed to the smallest nanoplastics (50 nm), in a dose-dependent manner. A study reported the same effect in other zooplankton species such as in *Daphnia pulex* [76]. In fact, the presence of nPS-NH_2_ aggregates in the digestive tract can limit food intake and significantly affect the growth and development of *A. salina* larvae. The sublethal toxicity of plastics, which is responsible for the occurrence of malformations, appears, therefore, to be related to size (dimensional toxicity). A careful review by Serrao and Marques-Santos [77] showed how sublethal adverse effects increase after the experimental transformation of microplastics to nanoplastics. It is also interesting to note that growth inhibition was discovered in larvae with intestinal constriction. Such malformation, to date, has not been found in the literature in relation to exposure to polystyrene nanoplastics larger than 50 nm. These results prompt us to investigate these findings further to understand the size range responsible for this malformation and whether the latter may be associated with malabsorption related to growth inhibition.

## 5. Conclusions

The purpose of the present study was to clarify the effects of polystyrene nanoplastics with diameters of 50 nm and 100 nm on the development of *A. salina*. Toxicity, which was greater for the smaller nanoplastics, was expressed in sublethal effects involving growth inhibition (shorter body), intestinal malformations (constrictions), and increased apoptosis. The negative consequences of nanoplastic uptake were related to damage to larval organs and tissues, leading to the development of abnormal adult individuals with growth retardation and a lowered resistance to environmental changes. In addition, the localization of particles in the head of larvae suggests an alteration of the nervous system. These findings, in addition to those already in the literature, help determine the range of sizes and concentrations that are hazardous to marine biota and beyond. In addition, given the bioaccumulation of particles in the organs and tissues of zooplankton species, the transfer to higher aquatic organisms cannot be ignored. Therefore, prevention and environmental protection programs must be used to also protect human health endangered by the consumption of contaminated seafood species.

## Data Availability

Original data are available upon request.
